# Remarkable rutin-rich *Hypericum capitatum* extract exhibits anti-inflammatory effects on turpentine oil-induced inflammation in rats

**DOI:** 10.1186/s12906-019-2680-8

**Published:** 2019-10-29

**Authors:** Anca D. Farcas, Augustin C. Mot, Cezara Zagrean-Tuza, Madalina Ticolea, Bogdan Sevastre, Muhittin Kulak, Radu Silaghi-Dumitrescu, Alina Parvu

**Affiliations:** 1 Department of Biology, Faculty of Biology and Geology, Babeș-Bolyai University, RO-400028 Cluj-Napoca, Romania; 2 Department of Biomolecular Physics, National Institute for Research and Development of Isotopic and Molecular Technologies, RO-400293 Cluj-Napoca, Romania; 3 Department of Chemistry, Faculty of Chemistry and Chemical Engineering, Babeș-Bolyai University, RO-400028 Cluj-Napoca, Romania; 4 Department of Pathophysiology, Faculty of Medicine, “Iuliu Hațieganu” University of Pharmacy and Medicine, RO-400012 Cluj-Napoca, Romania; 5 Department of Pathophysiology, Faculty of Veterinary Medicine, University of Agricultural Sciences and Veterinary Medicine, RO-400372 Cluj-Napoca, Romania; 6 Department of Herbal and Animal Production, Vocational School of Technical Sciences, Igdir University, Igdir, Turkey

**Keywords:** *Hypericum capitatum*, Hypericaceae, Rutin, Oxidative stress, Anti-inflammatory

## Abstract

**Background:**

Natural extracts with beneficial biological activities are nowadays of high interest, in various treatment or prophylaxis. *Hypericum capitatum* has been known for its curative effects for centuries and its extracts have become of interest due to their distinct activity among other Hypericaceae members. In this study, further light is aimed to be shed on the secondary-metabolites composition of *H. capitatum* extracts, using chromatographic techniques and Electron paramagnetic resonance profiles in alkaline medium. Considering that no previous works explored the anti-inflammatory activity of *H. capitatum*, here, an in vivo study is also designed in order to evaluate this property by assessing the impact of one of *H. capitatum* extracts in ameliorating turpentine oil-induced inflammation on rats and to quantify their blood antioxidants level.

**Methods:**

Chromatographic techniques and Electron paramagnetic resonance spectroscopy were used in order to describe the chemical profile in different parts of the plant. The in vivo study on turpentine-oil induced inflammation in rats included three doses of *H. capitatum* extract expressed in rutin concentration. Oxidative stress was measured using total oxidative status, total antioxidant capacity, oxidative stress index, 3-nitrotyrosine, nitric oxide, malondialdehyde, superoxide dismutase, catalase and the inflammatory response was evaluated by performing a complete blood cells count and C reactive protein.

**Results:**

The extract was remarkably rich in rutin; however, other polyphenolic-like minor components appeared important in explaining the observed biological properties. The tested extract prevents the increase of inflammation-induced white blood cell count, number of neutrophils, and serum nitric oxide, and did so in a dose-dependent manner, similarly to the positive control—diclofenac. In addition, the same extract appeared to be a good alternative to diclofenac to restore total oxidative status, thiobarbituric active reactive species, total proteins and C reactive proteins. Moreover, antioxidant enzymes such as catalase, superoxide dismutase and total serum thiol concentration were significantly increased by the tested extract.

**Conclusions:**

Due to its powerful reservoir rich in rutin, *H. capitatum* extract depicted its in vivo antioxidant and anti-inflammatory effects indicating it to be a good alternative to conventional drugs for oxidative stress protection.

## Background

*Hypericum capitatum* Choisy is part of the *Hypericum* genus, Hypericaceae family, and it is widely spread in temperate regions. *H. capitatum* is characterized by the presence of translucent secretory canals or cavities that contain plant biomarkers [1]. These plants have been widely used over the present territory of Turkey starting from antiquity, as treatments for various diseases. The *Hypericum* spp. have been regarded as having various medicinal effects like antidepressant, antiseptic, antispasmodic and antimicrobial, and were used for a very long time in external wounds healing and gastric diseases [2]. In folk medicine *H. perforatum*, also called St. John’s Wort, is one of the best known representative of the *Hypericum* genus, and it was believed to cure ulcerated burns, gastric and urogenital infections and others [3].

*H. capitatum* is native to Lebanon, Syria and Turkey and it is a perennial herb that can reach up to 50 cm. It has the genus-specific small amber glands on both stem and leaves, and its flower color vary from dark orange to crimson [4]. This species has recently gained attention. In the few recent studies, the antibacterial activities of cell-culture extracts of *H. capitatum* var. *capitatum* against *Bacillus cereus*, *Staphylococcus aureus*, *Branhamella catarrhalis*, *Clostridium perfringens* and *Candida albicans* have been reported. Furthermore, the extracts of the plant exhibited a slight antiretroviral activity towards HIV-I [5]. In vitro antioxidant capacity [2] analysis of *H. capitatum* var. capitatum extracts, in various solvents, recommend this plant as a natural source of free radical scavengers.

Recent phytochemical profiling of *Hypericum* spp. points to a rich content of secondary bioactive metabolites, consisting of essential oils, naphthodianthrones, flavonoids and others [6]. One of the main chemotaxonomic markers of *Hypericum* spp. is the existence of essential oils, secreted by specialized glands which contain hydrocarbons, monoterpenes and sesquiterpenes with good antimicrobial activity. The naphthodianthrones cause the dark color of some *Hypericum* representatives’ leaf glands and flowers, and have phototoxic effects. The most important naphthodianthrones are hypericin and pseudohypericin, which are localized in leaves and flowers, with narrower leaves showing a higher hypericin content. Pharmaceutically speaking, naphthodianthrones stand out as the most interesting compounds in *Hypericum* genus because they proved to possess antiretroviral effects by inhibiting virus absorption. Flavonoids, another class of compounds present in relative high concentration in *Hypericum* genus, display strong antioxidant activities [6, 7]. One of the most common flavonoid that can be found in many plant species, is rutin; its importance has even led to a temporary (now obsolete) annotation as “vitamin P” [8–11] Many studies have revealed that plant extracts and their polyphenolic compounds (flavonoids and phenolic acids) also possess a series of attractive properties, such as antioxidant, anti-inflammatory, anti-hypertensive, etc. [8, 12, 13].

Reactive oxygen species (ROS) and reactive nitrogen species (RNS) initiate signaling cascades of pro-inflammatory reactions multiplying the inflammasome and systemic stress [14]. It is well documented that large amounts of pro-inflammatory substances such as nitric oxide (NO) are produced, and that afterwards RNS will increase [15]. Post-translational modifications, such as nitration of protein tyrosine (Tyr), increase during oxidative inflammatory conditions too [15]. Hence, such post-translational changes can be used as markers for the inflammation associated changes. *Hypericum* species have been known for the anti-inflammatory, antioxidant and anti-depressant-like effects [16–18], but the mechanisms underlying these beneficial effects are not fully understood.

The present work is aimed at studying a variety of *Hypericum capitatum*, more precisely to map the phytoconstituents and to assay both in vitro and in vivo the antioxidant capacity of such extracts using various parts of the plant collected at different developmental stages of the plant including pre-flowering, full-flowering and post flowering. Its effect on acute inflammation was also determined.

## Methods

### Reagents

The reagents included in standard assay kits for colorimetric and kinetic methods (thiobarbituric acid, vanadium (III) chloride (VCl_3_), methanol, diethylether, xylenol orange, [o-cresosulfonphtalein-3,3-bis (sodium methyliminodiacetate)], ortho-dianisidinedihydrochloride (3,3′-dimethoxybenzidine), ferrous ammonium sulfate, hydrogen peroxide, sulphuric acid, hydrochloride acid, glycerol, trichloroacetic acid (TCA)) were obtained from BioMaxima S.A., Lublin, Poland. Standard samples of rutin, kaempferol, caffeic acid, p-coumaric acid, isoquercitrin, quercitrin, apigenin, chrisin and chlorogenic acid were purchased from Sigma, Germany (purity higher than 95%). Gallic acid and quercetin were obtained from Roth, Germany. Hypericin and hyperforin with a purity level higher than 95% were purchased from Cayman Chemical. All other chemicals and solvents used in the study were of analytical grade.

### Plant specimen

*H. capitatum* CHOISY var. *capitatum* CHOISY exhibits natural distribution in the south-eastern parts of Turkey. Along with the study, the plant materials used were collected during vegetation period (April–June) of the plant in 2015 in Kilis. A voucher specimen with K.A.1109 number has been deposited at Department of Biology, Faculty of Arts and Sciences, Kilis 7 Aralık University (Kilis, Turkey). Whole *H. capitatum* plant along with seeds samples were collected in various flowering stages: pre-flowering stage, flowering stage and post-flowering stage. The plants were portioned as follows: powdered seeds, whole aerial parts (stem + leaves + flowers) from both flowering and post-flowering stages, stem, leaves and flowers from flowering stage, root and stem from post-flowering stage as well as leaves and stem from the pre-flowering stage. All of these were grounded to fine powder and used to prepare extracts that were analyzed for phytochemical analysis and in vitro antioxidant capacity, where for the in vivo antioxidant capacity determination and inflammation tests just the whole aerial parts of the flowering plant were used for reasons explained bellow. For chemical analysis comparison, dried whole plant *H. perforarum* (St. John’s wort), the best known representative of this genus, also commercially available, was purchased from Dacia Plant (Romania), batch number 82284/31.07.2018.

### Extraction procedure

Quantities of 8–65 g of several ground dried powder plant parts were mixed with methanol (3 times the mass of the sample, in mL) for 4 h. Following this, the suspension was centrifuged for 20 min at 4500 rpm. The pellet was washed two times with methanol and centrifuged, and the collected supernatant was filtered using 0.20 μm filters. The clear extract was collected and vacuum dried at 40 °C. Then the dried quantity (see Table 1) was weighed and deposited at − 80 °C for further analysis. The dried extract was either dissolved in pure DMSO (for in vivo tests) or methanol for phytochemical and in vitro analysis.

**Table 1:** Numbering of the extracts and yield (w:w) of extraction

No.	Part of plant (development stage)	material mass (g)	dried extract (g)	yield (%)
1	Leaf (pre-flowering stage)	10.13	0.88	8.6
2	Stem (pre-flowering stage)	8.58	0.62	7.2
3	Leaf (flowering stage)	8.512	0.53	6.3
4	Stem (flowering stage)	24.08	0.47	2.0
4	Flower (flowering stage)	15.73	1.37	8.7
**6**	Whole aerial parts – stem, leaf, flower (flowering stage)	65.22	3.34	5.1
7	Stem (post flowering)	21.98	0.99	4.5
8	Root (post-flowering stage)	30.58	0.98	3.2
9	Whole aerial parts – stem, leaf, flower (post flowering stage)	21.65	1.17	5.4
10	Powdered seeds	25.64	2.13	8.3

### Chromatographic analysis

High-performance thin layer chromatography (HPTLC) analysis was performed on plates (20 × 10 cm with silica gel 60 F254, 5–7 μm, Merck, Darmstadt, Germany), which were eluted using a mobile phase consisting of ethyl acetate: formic acid: water (10,1.5:2, v/v/v). The elution was performed at room temperature, on a distance of 8 cm and using glass chromatographic chambers (Camag), which were pre-saturated with the mobile phase for 30 min. Two and ten μL samples of the standard ethanolic solutions (0.1 mg/mL each) and 8 μL from the extracts (10 mg/mL) were applied on the plate as 8 mm bands using the Linomat 5- Camag applicator, at 1.5 cm distance from the low edge of plate, with 50 nL/s speed. The developed plates were heated at 50 °C for about 3 min and then were sprayed with 0.5% di-phenylboronic acid aminoethyl ester dissolved ethanolic solution. Compound detection was obtained UV light at 366 nm. HPLC-DAD phytochemical analysis was carried out using an Agilent Technologies 1200 HPLC Series system (Waldbronn, Germany) equipped with an on-line vacuum degasser, quaternary pump, temperature-controlled sample tray, automatic injector, a column thermostat compartment and a DAD detector. The chromatographic separations were run on a Zorbax SB-C18 100 × 3.0 mm i.d., 3.5 μM particle at 30 °C. The injection volume was 8 μL (10 mg/mL, 0.2 μm filtered extract), and the flow rate was 1 mL/min. The optimum method consisted of a gradient elution using solvent A, 10 mM ammonium acetate pH 5 and solvent B as acetonitrile. The steps were as follows: 0–2 min 4% B, 2–12 min, from 4 to 50% B, 12–19 min from 50 to 100% B, 19–20 min isocratic at 100% B and 20–20.1 min back to 4%B where was kept until 22 min. The UV-Vis detection of the compounds has been accomplished using the DAD detector that measured the entire spectrum in 210–700 nm region, every 1 s and the chromatograms were monitored at 254, 300, 340 and 590 nm. As standards there were chlorogenic acid, p-coumaric acid, caffeic acid, rutin, isoquercitrin, quercitrin, quercetin, apigenin, kaempferol and chrisin, hypericin and hyperforin. The identification of the compounds was employed by both chromatographic retention time (with a 0.3 s as tolerance) and spectral similarities (higher than 99.9% was considered as positive) which were done by the built-in software. The limit of quantification was about 1.5 μg/mL, depending on standard. The calibration curves (10–350 μg/mL, *n* = 6) range with R^2^ > 0.999.

### EPR measurements, in vitro antioxidant activity evaluation and total phenolic content

Antioxidant EPR-based studies have been previously described [19]. In this experiment, for each extract assay a diluted solution was prepared by mixing 10 μl of each sample with 90 μl 96% ethanol in a 500 μl Eppendorf tube. 8 μl of 0.5 M NaOH were added, changing the color of the mixture to yellow; the mixture was rapidly transferred to a glass capillary and placed in a quartz tube for EPR analysis. For spectral shape comparison, four standards were chosen: rutin, isoquercitrin, chlorogenic acid and luteolin. All measurements were performed on a Bruker EMX Spectrometer with continuous wave at X band (~ 9 GHz) at room temperature with the following parameters: microwave power 10.62 mW, modulation amplitude 0.5 G, center field 3515 G, and sweep field of 50 G. For in vitro antioxidant activity evaluation, three analytical methods were employed, namely DPPH bleaching assay (DPPH), trolox equivalent antioxidant capacity assay (TEAC) and inhibition of β-carotene bleaching assay, according to previously published protocols [20]. The total phenolic content was determined by Folin-Ciocâlteu method, with some modifications, as previously described [20]. The results were expressed in rutin or gallic acid equivalents based on calibrations curve developed using the same protocol.

### In vivo anti-inflammatory and antioxidant effects evaluation

Adult albino Wistar rats weighing 150.80 ± 7.34 g were accommodated at the Establishment for Breeding and Use of Laboratory Animals of the “Iuliu Hațieganu” University of Medicine and Pharmacy in Cluj-Napoca, Romania. Rats were housed in standard polypropylene cages, in standard laboratory conditions (temperature 25 ± 1 °C, relative humidity 55 ± 5%, and 12 h light/dark cycle). The rats were allowed free access to standard granular diet and water ad libitum. All the procedures performed on laboratory animals, comply with the Directive 2010/63/EU, and Romanian national law 43/2014 for protection of animals used for scientific purposes. The project was approved by the Veterinary Sanitary Direction and Food Safety Cluj-Napoca (no. 19/13.12.2016). Animals were randomly divided into 7 groups (*n* = 5), as follows: negative control group (C), which received DMSO 4.7%, the extract vehicle; inflammation group (I); anti-inflammatory drug group treated with diclofenac (10 mg/kg b.w.); rutin group, (25 mg/kg b.w); groups treated with three *H. capitatum* extract (whole aerial parts – stem, leaf, flower, flowering stage) dilutions expressed in rutin concentration (12.5, 25 and 50 mg rutin in extract/kg b.w.). We chose to use a minimum number of animals per group, in order to avoid the to euthanize too many animals. All treatments were performed once daily by oral gavage. Excepting group C, on the 7th day of the experiment the inflammation was induced by intramuscular administration of turpentine oil (0.6 mL/Kg b.w). On the 8th day, animals were anesthetized by subjecting them to deep inhalator narcosis with 4% isoflurane and 1.5 L/min oxygen flow, so that venous blood may be collected from orbital plexus and serum was stored at − 80 °C until use. After the initial blood collection, the animals, still under anesthesia, were euthanized by cervical dislocation.

The total oxidative status (TOS) [21] and the total antioxidant capacity (TAC) were determined using a colorimetric assays [21]. The ratio between TOS and TAC representing the oxidative balance, known as oxidative stress index (OSI), was calculated [22]. The NO synthesis was indirectly evaluated by measuring serum nitrites and nitrates (NOx) using the Griess reaction [22]. For this test serum samples were previously passed through 10-kDd filters and contaminant proteins were removed by extraction with 3:1 (v:v) solution of methanol: diethyl ether (1,9; v:v) [23]. 3-Nitrotirosine (3NT) was measured by ELISA method, using an ELISA kit from SunRed Biotechnology Company, China. Lipid peroxides were quantified by measuring serum thiobarbituric active reactive species (TBARS) [24]. Total thiols (SH) were determined as previously described [24]. Catalase (CAT) activity was measured by a kinetic method, using H_2_O_2_ as substrate [25], and superoxide dismutase (SOD) was measured as previously described [26]. Inflammatory response was evaluated using the liver and bone marrow acute phase responses. The liver acute phase response was evaluated by measuring total proteins (TP) and C reactive protein (CRP) with commercial standard assay packages obtained from Biomaxima S. A (Lublin, Poland). The bone marrow acute phase response was evaluated by performing a complete blood cells count (CBC) using Abacus Junior Vet automatic analyzer, based on Coulter’s electrical impedance principle (Diatron Messtechnik, Budapest, Hungary).

### Statistical analysis

All data are reported as the mean ± standard error of the mean (SEM). The Gaussian distribution was checked by the Shapiro-Wilk normality test. One-way analysis of variance ANOVA, followed by Bonferroni’s Multiple Comparison test procedure was performed. Statistical significance was at *p* < 0.05 (95% confidence interval). Statistical values were obtained using GraphPad Prism version 5.0 for Windows, GraphPad Software, San Diego, California USA.

## Results

**Fig. 1:**
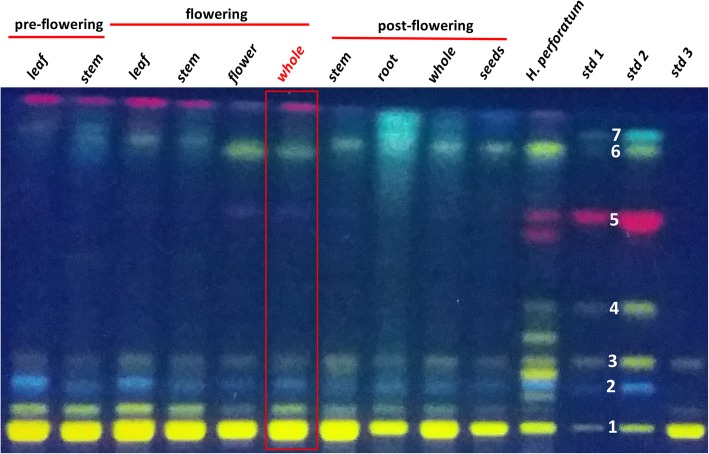
HPTLC analysis results indicating the main secondary metabolites contained by *Hypericum capitatum* extracts (each lane contains 8 μL of 10 mg/mL). Std1–2 μL and std. 2–10 μL of standards mixture containing rutin (1 – Rf = 0.07), chlorogenic acid (2 – Rf = 0.18), isoquercitrin (3 – Rf = 0.26), hyperoside (4 – Rf = 0.41), hypericin (5 – Rf = 0.67), quercetin (6 – Rf = 0.86) and kaempferol (7 – Rf = 0.89), all at 100 μg/mL. Std3 - standard of rutin solution – 2 μL from 3.5 mg/mL

A high-performance thin-layer chromatography (HPTLC) analysis was performed in order to preliminary evaluate the profile of main secondary metabolite composition of each extract. It is considered that this fast-analytical method can quickly provide informative data regarding the main composition concerned with polyphenols, phenylpropanes and eventually other compounds of the *H. capitatum* [6]. The compounds were firstly identified by their retention factor (R_f_) and then on their fluorescence color. In addition to the *H. capitatum* extracts, a mixture of standards including rutin, chlorogenic acid, isoquercitrin, hyperoside, hypericin, kaempferol and quercetin was also analyzed. These compounds were previously reported in other members of Hypericaceae family. A sample from a representative of the same family, the well-known *H. perforatum* (St. John’s wort) [1], was also included in HPTLC analysis to offer a reliable comparison agent for the studied plant. HPTLC results are shown in Fig. 1. Besides rutin, chlorogenic acid, isoquercitrin and kaempferol were also present, but in far smaller concentrations compared to rutin.

**Fig. 2:**
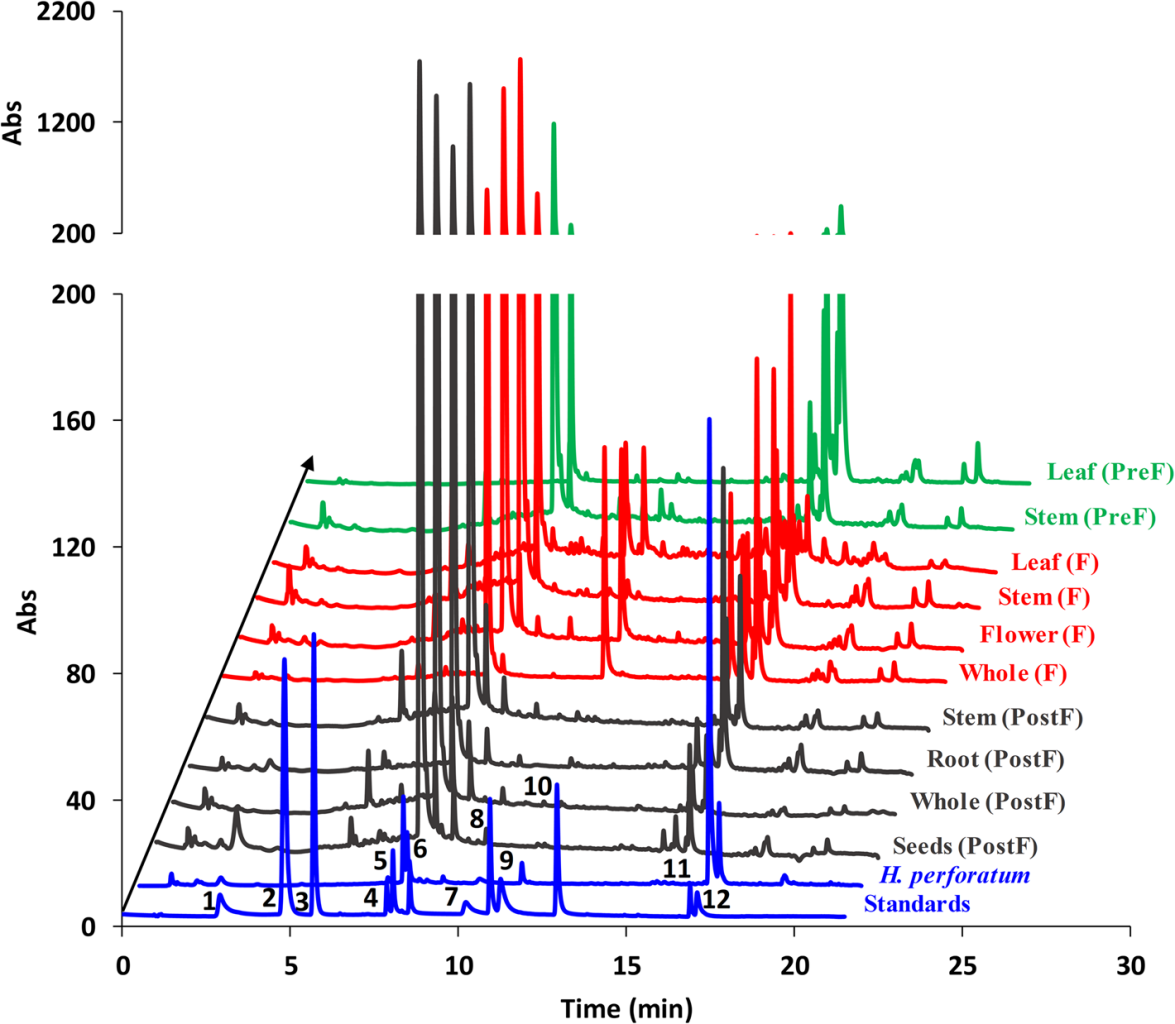
HPLC profiles of the studied *H. capitatum* extracts. Standards: 1 – chlorogenic acid, 2 – p-coumaric acid, 3 – caffeic acid, 4 – rutin, 5- isoquercitrin, 6 – quercitrin, 7 – quercetin, 8 – apigenin, 9 – kaempferol, 10 – chrysin, 11 – hyperforin, 11 – hypericin. By far, the highest chromatographic peak is rutin which accounts more than 70% peak area, in all cases. The y axis is rescaled and adapted for clarity

The same samples were analyzed by means of HPLC-DAD, which allows a highly resolved separation of components in a mixture, as well as a precise determination of each retention time. By means of HPLC-DAD it was possible not only to identify compounds based on their UV-vis spectra, but also to precisely quantify them. HPLC supported the use of an enriched standards mixture, containing caffeic acid and p-coumaric acid, along with chrysin and hyperforin, both being low-level phytoconstituents previously reported in literature in other Hypericaceae family members [6]. The corresponding chromatograms were plotted in Fig. 2, whereas Table 2 highlights the spotted compound along with the determined concentration and retention time. As can be observed from Fig. 1, all *H. capitatum* extracts, no matter what flowering stage or plant part was analyzed, exhibited a very high level of rutin. In fact, HPLC analysis pointed to impressively elevated levels of this glycoside, of hundreds or even thousands units as can be noticed in Table 2.

**Table 2:**
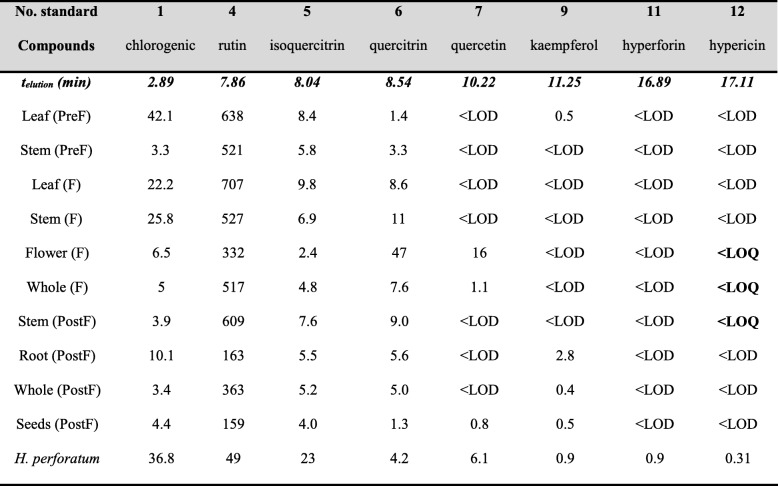
Elution time (in minutes), found concentrations (μg/mg, dried weight) for the tested standards in the studied samples for different parts of the *H. capitatum* at pre-flowering (PreF), flowering (F) and post-flowering (PostF) stages. The other standards (2, 3, 8, 10, Fig. 2) are all bellow LOD

Antioxidant activities of each prepared extract were analyzed by means of three methods along with total phenolic content. The results are shown in Table 3. The antioxidant capacity exhibited by extracts prepared from specific parts of the plant in a flowering stage (e.g. flower in flowering stage and leaves in pre-flowering stage) were higher than other values previously reported in the literature for other Hypericaceae family members, whereas the post-flowering stage whole aerial part extract accounts for the smallest determined activity.

**Table 3:**
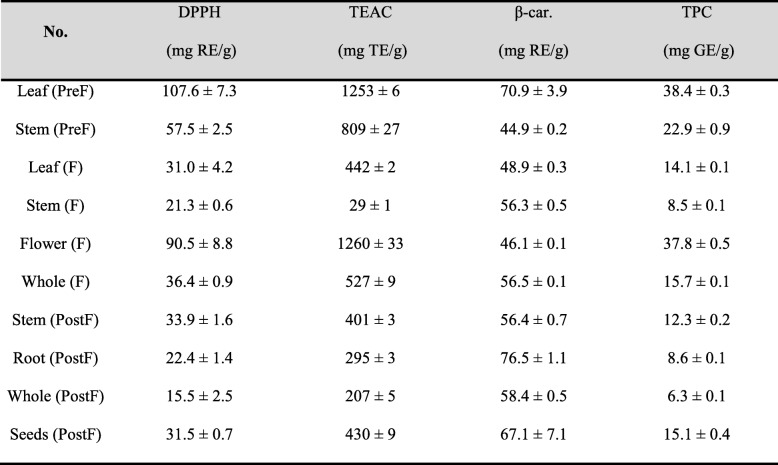
In vitro antioxidant activity evaluation using three distinct methods and total phenolic content (TPC). Results are expressed in rutin equivalents (RE), trolox equivalents (TE) and gallic acid equivalents (GE) in dried extract

Legend: *DPPH* DPPH decoloration assay, *TEAC* Trolox equivalent antioxidant capacity, *β-car. Bl.* β-carotene bleaching assay, *TPC* Total phenolic content determination by Folin-Ciocâlteu method. Extract legend described in Materials and Methods

**Fig. 3:**
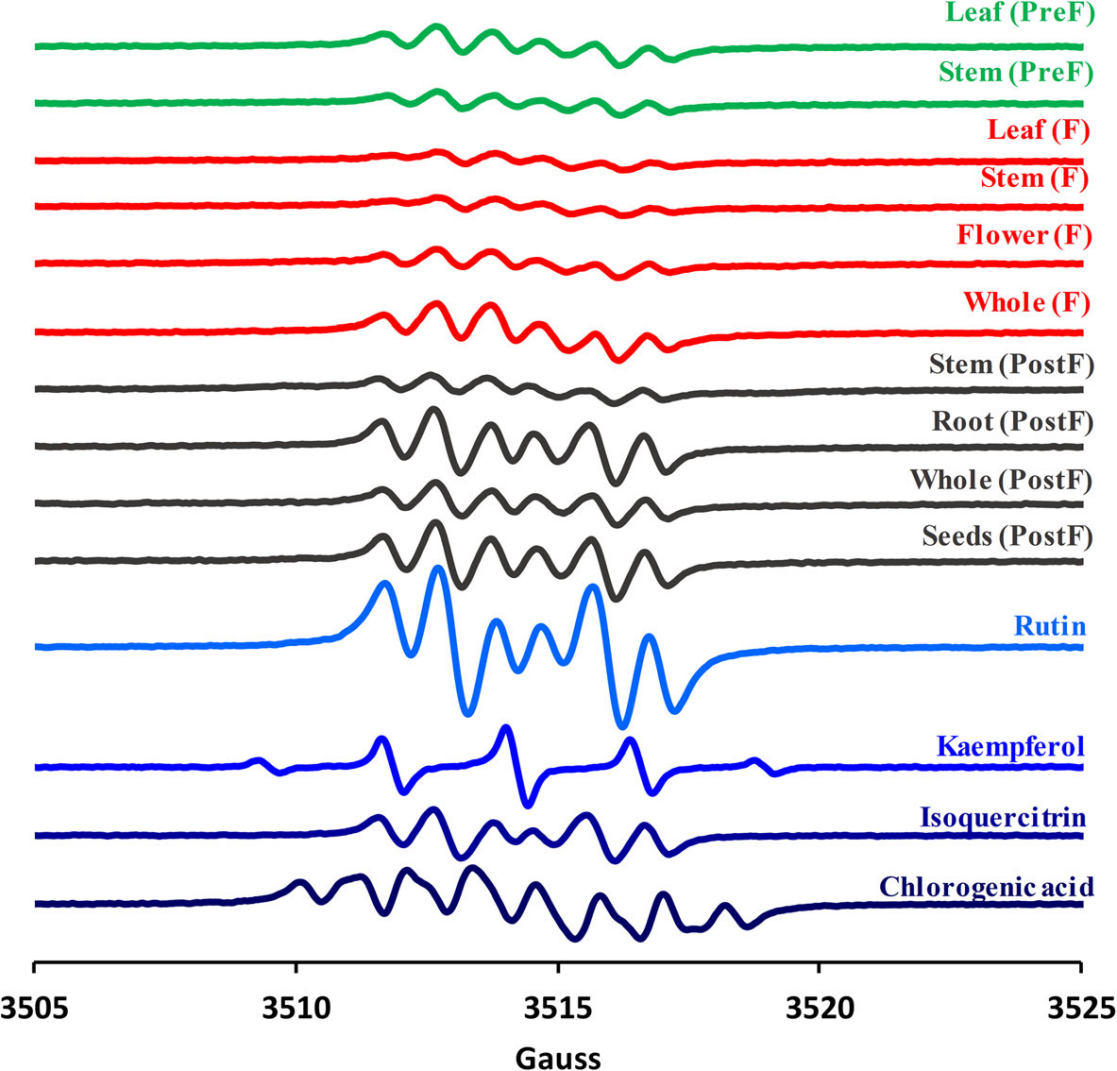
EPR spectra of all ten studied extracts of *H. capitatum* and four most representative standards

The EPR spectra of the 10 extracts are shown in Fig. 3. Besides, spectra of four representative standards were measured - namely rutin, isoquercitrin, cholorogenic acid and kaempferol, due to the fact that phytochemical analysis revealed their presence in considerable concentrations.

**Fig. 4:**
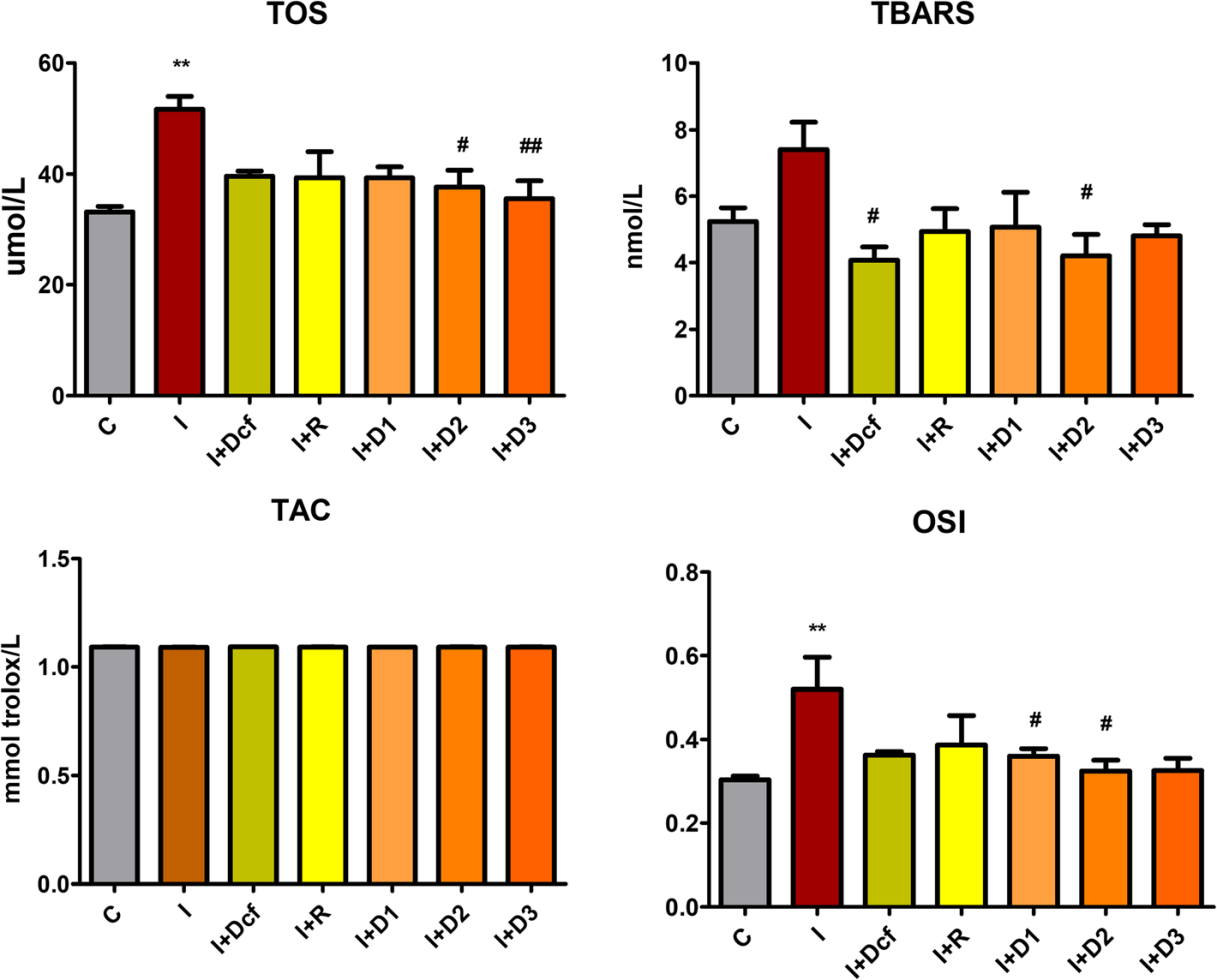
In vivo *antioxidant effects of H. capitatum extract on TOS, TBARS, TAC and OSI levels in serum*. Data represent mean ± SEM; One-way ANOVA followed by Bonferroni’s Multiple comparison test; * Significant at *p* < 0.05; **Significant at *p* < 0.01; ***Significant at *p* < 0.00 – compared with C group; ^#^ Significant at *p* < 0.05; ^##^ Significant at *p* < 0.01; Significant at *p* < 0.00 – compared with I group; *[C- Control; I- Inflammation; Dcf- Diclofenac; I + R- Inflammation + Rutin; I + D1 - Inflammation + Dose 1 of H. capitatum extract; I + D2 - Inflammation + Dose 2 of H. capitatum extract; I + D3 - Inflammation + Dose 3 of H. capitatum extract]*

Turpentine oil-induced inflammation is a model of acute inflammation [27]. In the present study, oxidative stress was evaluated using TOS, TAC and OSI tests as presented in Fig. 4.

**Fig. 5:**
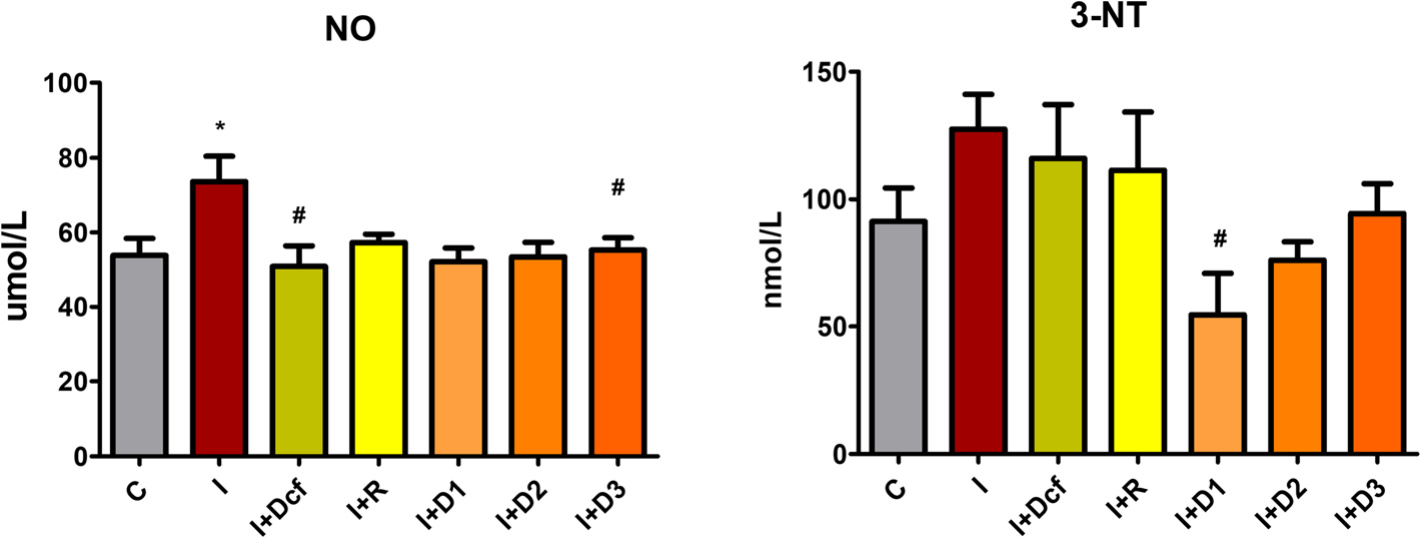
In vivo *anti-inflammatory effects of H. capitatum extract on NO and 3-NT levels in serum*. Data represent mean ± SEM; One-way ANOVA followed by Bonferroni’s Multiple comparison test; * Significant at *p* < 0.05; **Significant at *p* < 0.01; ***Significant at *p* < 0.00 – compared with C group; ^#^ Significant at *p* < 0.05; ^##^ Significant at *p* < 0.01; Significant at *p* < 0.00 – compared with I group;*[C- Control; I- Inflammation; Dcf- Diclofenac; I + R- Inflammation + Rutin; I + D1 – Inflammation + Dose 1 of H. capitatum extract; I + D2 - Inflammation + Dose 2 of H. capitatum extract; I + D3 - Inflammation + Dose 3 of H. capitatum extract]*

The NO synthesis was evaluated by measuring the serum concentrations of NOx and 3-NT as shown in Fig. 5. Inflammation increased NOx, as compared to C group (*p* < 0.5). All tested treatments had an inhibitory effect on NOx (*p* < 0.05) as compared to I group. The three doses of *H. capitatum* extract reduced NOx concentration almost to the control level and similar to diclofenac. The highest dilution of *H. capitatum* extract had the best inhibitory effect on NOx.

**Fig. 6:**
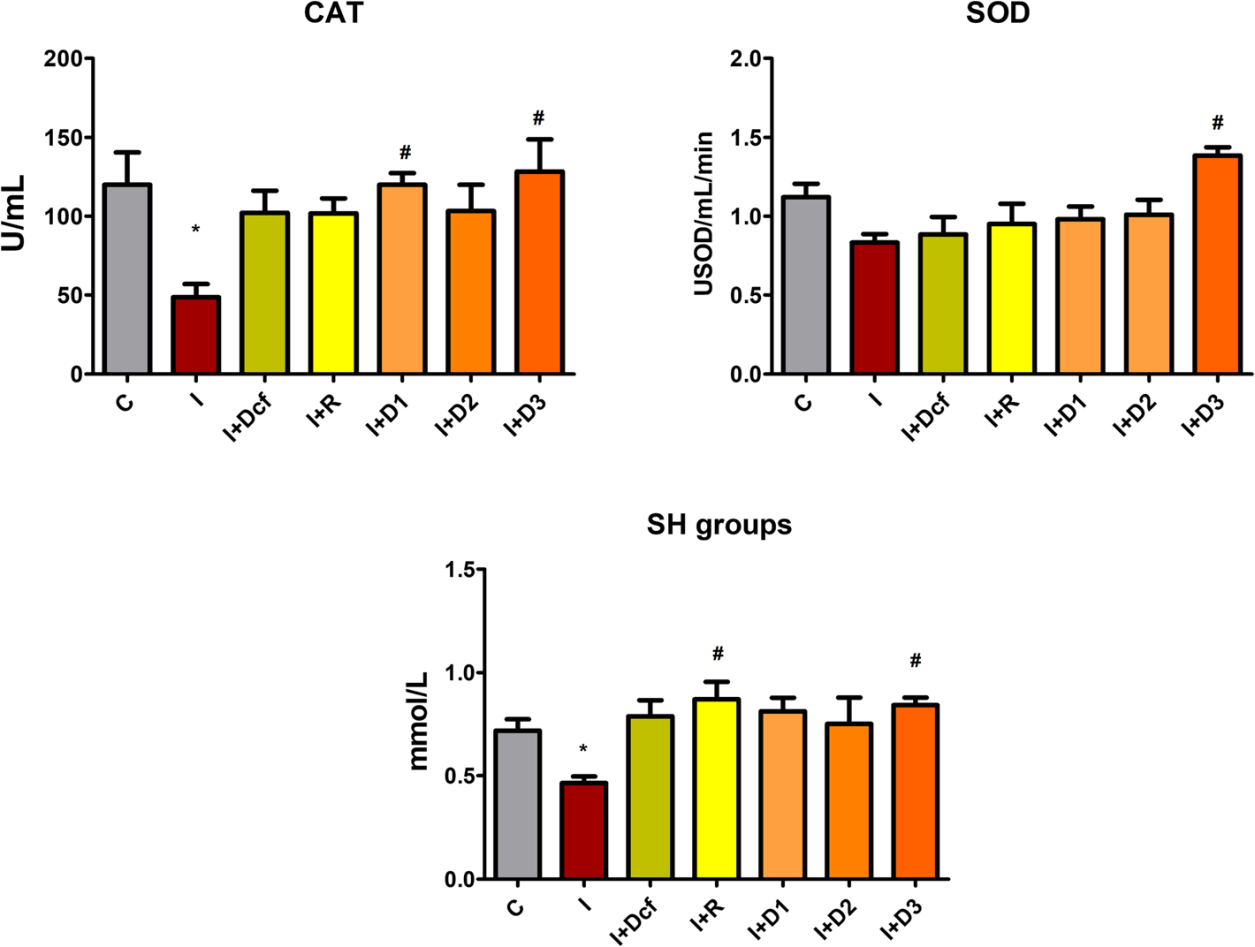
*Effects of H. capitatum extract on CAT and SOD activities, as well as SH concentration in serum.* Data represent mean ± SEM; One-way ANOVA followed by Bonferroni’s Multiple comparison test; * Significant at *p* < 0.05; **Significant at *p* < 0.01; ***Significant at *p* < 0.00 – compared with C group; ^#^ Significant at *p* < 0.05; ^##^ Significant at *p* < 0.01; Significant at *p* < 0.00 – compared with I group; *[C- Control; I- Inflammation; Dcf- Diclofenac; I + R- Inflammation + Rutin; I + D1 - Inflammation + Dose 1 of H. capitatum extract; I + D2 - Inflammation + Dose 2 of H. capitatum extract; I + D3 – Inflammation + Dose 3 of H. capitatum extract]*

As noticed in Fig. 6, almost all experimental pretreatments could not prevent SOD activity reduction by the acute inflammation, excepting D3 the highest extract concentration, which in fact managed to increase SOD activity above the control level (*p* < 0.05, though one may note that such increases reinforce the need to consider the anti-oxidant/pro-oxidant balance manifested by most antioxidant compounds and extracts [28].

As seen in Fig. 6, in the CAT graph, there is a significant decrease in the CAT activity in group I, which correlates with a reduction in SOD activity. CAT activity was increased to near-normal values by all tested substances.

The free/reduced thiol concentration, which is important in total antioxidant capacity, [10] was also investigated. Figure 6 reveals the levels of SH groups in all experimental groups. As expected, they decreased in the inflammation group (p < 0.05), whereas rutin increased their level, as well as the extracts, mostly D_3_ (*p* < 0.01), as compared to the I group. As seen in Table 4, the *H. capitatum* extract, mostly D_3,_ rutin and diclofenac effectively prevented the increase in WBC and NEU as compared to the I group.

**Table 4:** Complete blood count of control and experimental animals. Values are expressed as mean ± SEM

Parameters	Control	I	Diclofenac-I	R-I	D_1_-I	D_2_-I	D_3_-I
WBC _(10^9/L)_	7.53 ± 0.32	10.36 ± 0.58 ^*^	4.77 ± 0.62^#***^	5.43 ± 0.31^#***^	8.05 ± 0.55	6.57 ± 0.29^#**^	5.50 ± 0.93^#***^
LYM _(10^9/L)_	6.31 ± 0.79	6.46 ± 0.65	4.26 ± 0.44	4.58 ± 0.42	6.39 ± 0.24	5.76 ± 0.44	4.64 ± 0.23
MON _(10^9/L)_	0.61 ± 0.18	0.65 ± 0.16	0.46 ± 0.18	0.28 ± 0.08	0.52 ± 0.14	0.42 ± 0.09	0.38 ± 0.09
NEU _(10^9/L)_	1.48 ± 0.07	3.03 ± 0.14 ^**^	1.04 ± 0.08^#***^	1.63 ± 0.39^#*^	2.60 ± 0.59	1.96 ± 0.29	1.36 ± 0.13^#**^
RBC(_10^12/L_)	8.40 ± 0.37	9.11 ± 0.34	8.59 ± 0.16	8.66 ± 0.15	8.88 ± 0.17	8.61 ± 0.06	9.24 ± 0.14
HGB (g/dL)	14.20 ± 0.55	14.85 ± 0.36	14.64 ± 0.27	15.08 ± 0.27	14.88 ± 0.19	14.88 ± 0.36	15.26 ± 0.21
HCT_(%)_	43.78 ± 1.48	46.02 ± 1.57	44.98 ± 0.67	45.69 ± 1.09	45.74 ± 0.78	46.50 ± 0.92	46.67 ± 0.71
MCV_(fl)_	52.20 ± 1.39	49.20 ± 0.73	52.20 ± 0.58	52.60 ± 1.40	51.60 ± 0.74	52.00 ± 1.04	50.40 ± 0.87
MCH_(pg)_	16.92 ± 0.34	16.24 ± 0.28	17.06 ± 0.32	17.40 ± 0.32	16.74 ± 0.12	16.90 ± 0.46	16.52 ± 0.12
MCHC_(g/dL)_	32.44 ± 0.29	32.92 ± 0.35	32.56 ± 0.36	32.98 ± 0.28	32.56 ± 0.43	32.56 ± 0.61	32.72 ± 0.36
RDWs_(%)_	17.04 ± 0.21	17.20 ± 0.27	17.04 ± 0.11	16.62 ± 0.20	17.00 ± 0.20	17.18 ± 0.28	17.24 ± 0.39
PLT_(10^9/L)_	737.8 ± 190.8	944 ± 94.61	800.4 ± 61.83	840 ± 50.71	902.4 ± 65.56	840.2 ± 25	784.4 ± 67.7
PCT_(%)_	0.51 ± 0.13	0.65 ± 0.05	0.54 ± 0.03	0.57 ± 0.03	0.62 ± 0.04	0.57 ± 0.02	0.53 ± 0.06
MPV_(fl)_	6.92 ± 0.09	6.98 ± 0.15	6.80 ± 0.20	6.88 ± 0.20	6.90 ± 0.04	6.88 ± 0.13	6.78 ± 0.15
PDWs_(%)_	33.54 ± 0.49	32.78 ± 0.33	32.08 ± 0.23	33.08 ± 0.32	33.24 ± 0.20	32.76 ± 0.46	32.58 ± 0.42

[*WBC* White blood cell count, *NEU* the Number of neutrophils, *MON* Monocytes, *LYM* Lymphocytes, *RBC* Red blood cells count, *HGB* Haemoglobin concentration, *HCT* Haematocrit, *MCH* Mean corpuscular haemoglobin, *MCV* Mean corpuscular volume, *MCHC* Mean corpuscular haemoglobin concentration, *RDW* Red blood cell distribution width, *PLT* Platelet count, *PCT* Thrombocytocrit, *MPV* Medium platelet volume and *PDW* Platelet distribution width]

* Significant at *p* < 0.05; ** Significant at *p* < 0.01; *** Significant at *p* < 0.001– compared with C group; ^#^ Significant at *p* < 0.05; ^##^ Significant at *p* < 0.01; Significant at *p* < 0.001 – compared with I group

In order to complete the picture of the systemic acute phase response, serum proteins and CRP concentrations were also evaluated. As seen in Table 5, in the I group the total protein content, alongside with globulins and CRP concentrations were significantly elevated (*p* < 0.05) as compared to the C group.

**Table 5:** Plasma proteins and C reactive protein (CRP) of control and experimental animals. Values are expressed as mean ± SEM

Parameters	Control	I	Diclofenac-I	R-I	D_1_-I	D_2_-I	D_3_-I
TP_(g/dL)_	7.60 ± 0.97	10.42 ± 0.54^a*^	9.87 ± 0.66	10.14 ± 0.31	9.46 ± 0.26	8.80 ± 0.38	9.55 ± 0.60
ALB _(g/dL)_	2.11 ± 0.22	2.8 ± 0.86	2.60 ± 0.80	2.53 ± 0.85	2.75 ± 0.56	2.75 ± 0.59	2.59 ± 0.7
GLOB _(g/dL)_	5.19 ± 0.42	7.62 ± 0.54 ^a*^	7.11 ± 0.32	7.05 ± 0.42	6.61 ± 0.24	6.05 ± 0.32	6.86 ± 0.20
CRP _(mg/L)_	39.20 ± 2.47	73.25 ± 19.19 ^a*^	42.0 ± 6.38	37.0 ± 13.81	38.40 ± 8.17	21.40 ± 8.17^#*^	23.80 ± 6.05^#*^

[*TP* Total proteins, *ALB* Albumin, *GLOB* Globulins, *CRP* C reactive proteins]

* Significant at *p* < 0.05; ** Significant at *p* < 0.01; *** Significant at *p* < 0.001– compared with C group; ^#^ Significant at *p* < 0.05; ^##^ Significant at *p* < 0.01; Significant at *p* < 0.001 – compared with I group

^a^ compared with Control

## Discussions

It was previously reported that the Hypericaceae family contains flavonol glycosides of quercetin in their aerial parts, especially in rutin, however, *H. capitatum* appears to exhibit much more rutin than the well-known *H. perforatum*. There are specific stages in plant development when there are high concentrations of rutin namely at the budding stage or the immediate post-flowering stage [6]; this is supported by the HPTLC data, where the extract corresponding to the whole aerial parts of the plant in the post-flowering stage yielded one of the largest and most intense spots. On the other hand, as seen in Table 2, the specific individual aerial parts of the plant are the richest in rutin, especially leaves and stems, in all flowering stages. Interestingly, previous literature studies pointed out that *H. perforatum* flowers are the richest in this glycoside [29].

The presence of isoquercitrin and hyperoside is also a biomarker of this genus [6, 7]; in the case of the studied *H. capitatum*, only isoquercitrin was present in detectable amount, and at far smaller concentration compared to that determined for rutin. It has been outlined before that, when it comes to *Hypericum* species, there is a positive correlation between rutin concentration and altitude of plant environment, whereas there is a negative dependence if isoquercitrin levels are considered [29]. Quercitrin has also been identified and quantified as a result of HPLC analysis, exhibiting higher concentrations than isoquercitrin. Also, a small amount of quercetin has been determined, which is believed to come from the hydrolysis of its glycosides [6]. In fact, quercetin and other aglycones concentration is generally too small in this family to attribute the antioxidant capacity of this plants to their presence. The presence of kaempferol in detectable amounts is also remarkable, considering the fact that few other representatives of this family contain this polyphenol. Interestingly, most of the aerial parts of the plant do not exhibit this flavonoid. Chlorogenic acid is also present in small concentrations as an exponent of phenylpropanes, another specific biomarker for this genus, whereas p-coumaric and caffeic acids were not identified by HPLC. All this metabolite profile is similar to the one of St. John’s wort, except the fact that latter presents another flavonoid glycoside. A truly remarkable aspect that seems to differentiate *H. capitatum* from its relatives is the low level of hypericins, detectable only in traces in flowering period and lack of other specific phytoconstituents, most notably chrysin and hyperforin. The most specific biomarker of this family, the naphthodianthrones, are thought to be responsible for the therapeutic effects of the *Hypericaceae* family - and such compounds can be found in all well-studied exponents. Interestingly, they are present in low levels in *H. capitatum*. In previous studies, there were some situations when naphthodianthrones could not be identified in Hypericaceae family due to their small concentration; apparently, during winter the levels of hypericins are smaller than 100 ppm and can be hardly quantified by use of classical methods, whereas in summer they are up to 3000 ppm. The reason for this change in concentration is still unknown. Another experiment points out the fact that hypericins levels can be inversely proportional to the concentration of xanthones, other family of specific secondary metabolites of Hypericaceae family produced in dominant quantity in in vitro plant cells culture, even though they are in far smaller concentration in their in vivo parents [6].

It can be noted that by far the antioxidant activities of extracts prepared from flowers in the flowering stage and leaves in the pre-flowering stage were the highest in all samples analyzed. This might be correlated with an intense photosynthesis and some metabolic changes in various plant flowering stages, considering the fact that secondary metabolites serve as both signaling and antioxidant molecules. TEAC should be considered a measure for total antioxidant capacity [30]. On the other hand, total phenolic content determination by Folin-Ciocâlteu method revealed high amounts of polyphenols. There is a very good correlation between TEAC assay, DPPH bleaching assay and total phenolic content determined by Folin-Ciocâlteu method [30, 31]. When it comes to β-carotene bleaching assay, there is no correlation between its results and those from other methods. This may be due to the difference in mechanism between β-carotene bleaching method, based on hydrogen atom transfer, and DPPH bleaching and TEAC assays (mainly single electron transfer).

Along with the HPTLC/HPLC analysis and in vitro antioxidant determination, EPR tests revealed the same aspect: by using the extraction protocol described before, the secondary metabolite present in the highest concentration was by far rutin and this dominates the reactivity of the extract. As one can observe, EPR spectra of all ten *H. capitatum* extracts were very similar in rutin profile, pointing to its dominant contribution in EPR spectrum. Still, minor influences of other compounds (e.g., as chlorogenic acid and kaempferol) can also be observed EPR spectra of the extracts. *H. capitatum* flowers appear to have the highest antioxidant activity and the richest in terms of phytochemical compounds (numerous chromatographic peaks). However, flowers alone have limited biomass; therefore, whole aerial parts during flowering stage were used for biological tests. Moreover, this is usually the case of the commercial *Hypericum* tea.

TOS is a marker of total oxidants, and it is an extremely useful tool when measuring the efficiency of plant extracts [32]. Preventive treatment with diclofenac, rutin and *H. capitatum* extract reduced TOS, to almost normal values. In groups I + D2 (*p* < 0.05) and I + D3 (*p* < 0.01) *H. capitatum* had a better inhibitory effect on TOS than rutin, indicating involvement of the flavonoids from the extract. The TAC assay, which generally allows for a global evaluation of serum antioxidants, did not reveal significant differences between the experimental groups, suggesting no changes in the general concentration of antioxidants by any of the treatments and pre-treatments. The OSI expectedly followed the same pattern as TOS. It was previously demonstrated that rutin has a dose-dependent inhibitory effect in inflammation [33]. During oxidative-inflammatory conditions NO, also induces protein tyrosine (Tyr) nitration and 3-nitrotyrosine (3-NT) formation [15, 34]. Therefore, the highest 3-NT is associated with the inflammation group. The *H. capitatum* extract treatments had an inverse dose-dependent inhibitory effect on 3-NT formation, D1 having the strongest inhibitory effect and D3 the lowest. Because in I + R group there was no significant change of 3-NT, it was considered that other antioxidant compounds from the extract could have an important contribution 3-NT reduction. SOD activity depends on several different factors, mostly when it comes to in vivo. Firstly, during inflammation, nitration can impair the activity of antioxidant enzymes such as SOD [35], to the extent that in vivo SOD will have half the superoxide scavenging activity [36]. Secondly, when released in excess, NO reacts with superoxide (•O_2_) leading to peroxynitrite (ONOO^−^) formation, a transient RNS that can affect a large range of biomolecules [35, 37]: peroxynitrite may react with SOD leading to the formation of ONOO^−^ -SOD adduct [15] causing SOD efficiency reduction. The management of intracellular oxidative stress relies on maintaining a balance in the activities of the SOD and CAT. D_1_ (*p* < 0.05) and D_3_ (*p* < 0.05) proved again the efficacy of the *H. capitatum* extract.

This extract works efficiently by quenching reactive free radicals [38, 39]. Protein thiol groups are involved in plasma antioxidant reactivities well as in signaling and antioxidant defense and catalysis [40]. Even though inflammation starts as a local reaction, it associates a systemic acute phase response [41] that involves pro-inflammatory mediators (IL-6; IL-1β; TNF-α), bone marrow response causing leukocytosis, liver response with acute phase proteins (APPs) synthesis [27, 41]. Both diclofenac and the extract (D_1_, D_2_ and D_3_) decreased plasma proteins and C reactive protein levels, almost to the control values. Rutin reduced only CRP. Taken together, these results indicate that the inhibitory effect of *H. capitatum* on the systemic acute phase responses also depends on other minor components from the extract, in addition to rutin.

## Conclusions

To conclude, an extensive study on secondary metabolites has been carried out on various development stages of *H. capitatum* extracts, by means of HPTLC and HPLC, revealing distinctly high levels of rutin, and minor content of chlorogenic acid and kaempferol and only traces of hypericins (only in flowering period). The EPR signal shape of all ten tested extracts was highly influenced by the elevated concentrations of rutin. In vivo*, pretreatment* with *H. capitatum* extracts was found to reduce inflammation-induced oxidative stress by lowering the ROS and RNS, plus by increasing some endogenous nonenzymatic and enzymatic antioxidants, in a dose-dependent manner, similarly as diclofenac. These results encourage future studies in order to develop plant-derived alternatives for conventional anti-inflammatory drugs such as diclofenac.

## Data Availability

All data described in the manuscript are available from the corresponding author on reasonable request.

## Abbreviations

3NT: 3-nitrotyrosine
ALB: Albumin
APPs: Acute phase proteins
CAT: Catalase
CBC: Complete blood cells count
CRP: C reactive protein
DPPH: 2,2-Diphenyl-1-picrylhydrazyl or 2,2-diphenyl-1-picrylhydrazyl bleaching assay
EPR: Electron paramagnetic resonance spectroscopy
GLOB: Globulins
HCT: Haematocrit
HGB: Haemoglobin concentration
LYM: Lymphocytes
MCH: Mean corpuscular haemoglobin
MCHC: Mean corpuscular haemoglobin concentration
MCV: Mean corpuscular volume
MDA: Malondialdehyde
MON: Monocytes
MPV: Medium platelet volume
NEU: Number of neutrophils
NO: Nitric oxide
NOx: Serum nitrites and nitrates
OSI: Oxidative stress index
PCT: Thrombocytocrit
PDW: Platelet distribution width
PLT: Platelet count
RBC: Red blood cells count
RDW: Red blood cell distribution width
RNS: Reactive nitrogen species
ROS: Reactive oxygen species
SEM: Standard error of the mean
SOD: Superoxide dismutase
TAC: Total antioxidant capacity
TBARS: Thiobarbituric active reactive species
TCA: Trichloroacetic acid
TEAC: Trolox equivalent antioxidant capacity assay
TOS: Total oxidative status
TP: Total proteins
WBC: White blood cell count
